# Exoscopic resection of giant olfactory groove meningioma

**DOI:** 10.3171/2023.10.FOCVID23125

**Published:** 2024-01-01

**Authors:** Francesco Calvanese, Anna Maria Auricchio, Martin Lehecka

**Affiliations:** 1 Department of Neurosurgery, Helsinki University Hospital and University of Helsinki, Finland; and; 2 Department of Neurosurgery, University Hospital Foundation A. Gemelli IRCCS, Catholic University of the Sacred Heart, Rome, Italy

**Keywords:** exoscope, olfactory groove meningioma, lateral supraorbital approach, translational and angular movements, foot pedal

## Abstract

Olfactory groove meningiomas represent 8%–13% of all intracranial meningiomas. Gross-total resection for large (4–6 cm) and giant (> 6 cm) cases remains challenging due to their relationship with critical neurovascular structures and extensive frontal lobe edema. A variety of transcranial and endoscopic approaches have been described. This 2D operative video shows the use of a digital 3D exoscope in the removal of a giant olfactory groove meningioma through a lateral supraorbital approach in a 57-year-old woman with visual impairment and apathy. The exoscope provides a very good angulated view of the subfrontal area on both sides of the anterior cranial fossa even through a small craniotomy.

The video can be found here: https://stream.cadmore.media/r10.3171/2023.10.FOCVID23125

## Transcript

This 2D operative video shows the use of a digital 3D exoscope in the removal of a giant olfactory groove meningioma through a lateral supraorbital approach.

### 0:30 Clinical History.

The patient is a 57-year-old woman with a 12-month history of progressive visual field impairment and decreased vision of the right eye, anosmia, and apathy. The MRI shows a giant olfactory groove meningioma with an extensive frontal lobe compression and edema. On the T2 coronal imagines, we can see that the A1-ACom complex is displaced cranially to the right side, while the optic chiasm is shifted inferoposteriorly. The lesion appears vascularized, with the main feeders coming from ethmoidal arteries through the skull base at the level of the crista galli and extensive network of enlarged veins on the tumor surface. There are areas where arachnoid plane can be seen between the tumor and the brain, so at least some parts of the tumor surface will not be completely attached to the pial surface. Pericallosal arteries and some of their branches are running along the posterior and superior surface on the tumor.

### 1:27 Surgical Planning and Selection of the Exoscopic Corridors.

Even for giant anterior skull base tumors, we prefer to use small frontolateral approaches instead of large bifrontal approaches since they are faster to perform, they provide good exposure of all the necessary structures, and frontal sinuses are left intact. When choosing the side of the frontolateral approach, several factors must be considered. In this case, more of the tumor mass is located on the left side of the midline. This would favor a left-sided approach. However, the asymmetry and inclination of the anterior skull base at the crista galli region is such that the right-sided approach will expose the main tumor feeders for early devascularization much better than what a left-sided approach would offer. Also, the pericallosal arteries and the ACom complex are displaced to the right, so they will be easier to control from the right-sided approach. Moreover, the already existing visual impairment of the right eye is also in favor of the right-sided approach.

### 2:24 Patient Positioning and Craniotomy.

A right-sided lateral supraorbital approach was selected for this case. The patient’s head is fixed in a Sugita head frame, rotated about 50° to the opposite side and tilted laterally about 10°. After minimal shaving, a curvilinear skin incision is made at the hairline running from the midline toward the ear, stopping about 4 cm cranial to the origin of the zygoma. A small part of the anterocranial part of the temporal muscle is incised and turned frontobasally. One burr hole is planned under the temporal muscle. Medial and lateral craniotome cuts are connected by a groove along the frontobasal part close to the floor of the anterior fossa. The bone flap of about 4 cm is then cracked along this groove and elevated. High-speed drill is used to reach the floor of the anterior fossa and to flatten the sphenoid ridge at the lateral edge of the craniotomy. The dura is cut in a curvilinear fashion and tack-up sutures are placed to prevent epidural oozing.

### 3:27 Surgical Trajectories and Advantages of the Exoscope.

Intradurally, the first step will be tumor devascularization and detachment from the anterior skull base.

On the left-sided image, the yellow area represents the main area of the tumor attachment, and the red dotted line the area of bony hyperostosis. The blue area is the crista galli with falcine attachment colored in green.

The hyperostosis on the preoperative images extended from the crista galli posteriorly to the planum sphenoidale, especially on the left side of the anterior skull base.

The image on the right shows how even a rather small frontolateral craniotomy provides wide exposure at depth due to the keyhole effect.

This next image demonstrates how all the targeted areas can be reached and properly visualized by translational and angular movements of the exoscopic camera. This is one of the greatest advantages of the exoscope compared to a surgical microscope. All these different viewing angles can be achieved only by movement of the camera without any positional changes of either the surgeon or the patient.

The OR setting and the ergonomics of the surgeon are shown here.

### 4:33 Tumor Devascularization.

After introducing the exoscope (Aesculap Aeos), we move subfrontally toward the tumor attachment. We can immediately see a clear borderline between the tumor and brain. We follow the floor of the anterior fossa and progressively coagulate and cut the tumor attachment. The first aim is to reach the ipsilateral optic and carotid cisterns. Here we can on one hand secure the right optic nerve, but also we can open the basal cisterns to release CSF for gaining additional space.

The dissection is then oriented more medially as demonstrated by the blue cone. The next target area is the crista galli and the hyperostotic bone, where the main feeders of the tumor are.

The tumor at this region is detached by coagulation. The problem are arterial feeders coming directly through the bone as they cannot be coagulated by bipolar forceps. They are best handled by drilling with a fine diamond drill, so-called hot drilling, which coagulates these feeders by heating the bone locally.

We prefer not to drill the whole area of the hyperostosis on the anterior skull base but only the region where the main feeders are. Based on our experience, this reduces the risk of CSF leak due to accidental entering into the ethmoidal sinuses. At the same time, it does not seem to dramatically increase the risk of tumor recurrence.

Once the main feeders are taken care of, we move more anteriorly toward the proximal attachment of the falx as highlighted by the green cone.

The inferior part of the falx is coagulated and cut with microscissors. Care is taken not to enter into the superior sagittal sinus. This move allows us to reach the most anterior area of the contralateral anterior cranial fossa and the tumor attachment, as shown by the red cone. We continue by detaching the contralateral part of meningioma from the skull base. Our experience is that in the giant olfactory groove meningiomas, the olfactory nerves are already so damaged and thinned that there is no real chance of leaving them intact during the tumor removal.

The devascularization along the anterior skull base is progressively continued. The dissection is next oriented toward the most posterior part of the tumor attachment and the contralateral optic nerve. Keeping both hands in the operative field while rotating and moving the exoscope camera remotely by a foot pedal speed up the surgery and gives good control over the dissection. The excellent rotational possibility of the exoscope camera allows to visualize all the deeper regions of anterior skull base, respecting surgeon ergonomic even at extreme angles. At the same time, the improved magnification and 3D visualization give a better control of the entire surgical area and demonstrate the anatomy also for teaching purpose.

Once the whole tumor insertion is detached, we can see also the contralateral optic nerve.

Finally, the tumor is devascularized also from the small feeders coming from the falx at the anterosuperior region near the midline.

### 7:51 Tumor Dissection From the Brain Parenchyma.

With most of the tumor feeders gone, the next main goal is to start working on the tumor borderline. We use water dissection technique to expand the natural plane between the tumor and the brain, followed by blunt and sharp dissection to progressively detach the tumor from the pial surface.

All the arteries on the tumor surface must be dealt with very carefully. Some of them are en passant types; others are just feeders of the tumor. We prefer to dissect and save all the arteries initially. Only later, if we are very sure that they are only feeding the tumor, they can be coagulated and cut. Ultrasonic aspirator can be helpful in debulking and shrinking of the superficial part of the tumor for additional space along the tumor borderline.

### 8:40 Tumor Debulking and Removal.

In a very large tumor such as this, the next step is to progressively debulk the tumor to reduce its volume and allow for further dissection along the borderline. Since the tumor was previously devascularized, it is bleeding very little. The ultrasonic aspirator can be helpful in the debulking.

Once the tumor volume has been sufficiently reduced, it is possible to continue with the final dissection along the tumor borderline.

The last part of the meningioma is carefully dissected from the ipsilateral pericallosal artery.

Later, the more distal part of the falx is incised so that the superior part of the tumor on the contralateral side can be reached as well. Finally, the remaining tumor mass is removed.

### 9:41 Hemostasis and Closure.

After tumor removal, intraoperative anatomy can be appreciated. We can see the displaced ACom complex and the pericallosal arteries, the ipsilateral and contralateral optic nerves, and the contralateral carotid artery. Careful hemostasis is performed throughout the whole resection cavity. The closure is done in multilayer fashion.

### 10:05 Outcome.

There were no new postoperative deficits. At 3 months’ follow-up, the visual acuity as well as the visual fields had shown improvement. The gross-total resection was confirmed with MRI at 3 months.

### 10:18 References.

Additional papers discussing this topic.^1–5^

## Disclosures

The authors report no conflict of interest concerning the materials or methods used in this study or the findings specified in this publication.

## Author Contributions

Primary surgeon: Lehecka. Assistant surgeon: Calvanese, Auricchio. Editing and drafting the video and abstract: all authors. Critically revising the work: all authors. Reviewed submitted version of the work: all authors. Approved the final version of the work on behalf of all authors: Calvanese. Supervision: Lehecka.

## Supplemental Information

### Patient Informed Consent

The necessary patient informed consent was obtained in this study.
